# Association between willingness to use an overdose prevention center and probation or parole status among people who use drugs in Rhode Island

**DOI:** 10.1186/s12954-024-00969-0

**Published:** 2024-02-29

**Authors:** Michael Tan, Carolyn Park, Jacqueline Goldman, Katie B. Biello, Jane Buxton, Scott E. Hadland, Ju Nyeong Park, Susan G. Sherman, Alexandria Macmadu, Brandon D. L. Marshall

**Affiliations:** 1 Department of Epidemiology, Brown University School of Public Health, 121 South Main Street, Providence, RI 02903 USA; 2 School of Population and Public Health, University of British Columbia, Vancouver, BC Canada; 3 Department of Pediatrics, Harvard Medical School, Boston, MA USA; 4 Department of Health, Behavior, and Society, Johns Hopkins Bloomberg School of Public Health, Baltimore, MD USA

**Keywords:** Harm reduction, People who use drugs, Overdose prevention center, Willingness, Probation/parole, Criminal legal system involvement

## Abstract

**Background:**

Overdose prevention centers (OPCs) are being implemented in the United States as a strategy to reduce drug-related mortality and morbidity. Previous studies have suggested that people who use drugs (PWUD) with a history of criminal legal system (CLS) involvement (e.g. current probation/parole) are at greater risk of overdose but may also encounter significant barriers to OPC use. The objective of this study was to explore the association between willingness to use an OPC and probation/parole status in a sample of PWUD in Rhode Island.

**Methods:**

This study utilized data from the Rhode Island Prescription and Illicit Drug Study, which enrolled adult PWUD from August 2020 to February 2023. We used Pearson’s chi-square and Wilcoxon rank-sum tests to assess bivariate associations between willingness to use an OPC and probation/parole status (current/previous/never), as well as other sociodemographic and behavioral characteristics. In multivariable Poisson analyses, we examined the association between willingness to use an OPC and probation/parole status, adjusting for key sociodemographic and behavioral characteristics.

**Results:**

Among 482 study participants, 67% were male, 56% identified as white, 20% identified as Hispanic/Latine, and the median age was 43 (IQR 35–53). Nearly a quarter (24%) had never been on probation/parole, 44% were not currently on probation/parole but had a lifetime history of probation and parole, and 32% were currently on probation/parole. Most participants (71%) reported willingness to use an OPC, and in both bivariate and multivariable analyses, willingness to use an OPC did not vary by probation/parole status. Crack cocaine use and lifetime non-fatal overdose were associated with greater willingness to use an OPC (all *p* < 0.05).

**Conclusions:**

These data demonstrate high willingness to use OPC among PWUD in Rhode Island regardless of CLS-involvement. As OPCs begin to be implemented in Rhode Island, it will be imperative to engage people with CLS-involvement and to ensure access to the OPC and protection against re-incarceration due to potential barriers, such as police surveillance of OPCs.

## Introduction

The criminalization of drug possession has had far-reaching impacts on people who use drugs (PWUD), including higher rates of incarceration and criminal legal system (CLS)-involvement, especially for Black and Brown communities who experience over-policing in the United States [1, 2]. Among those who are CLS-involved, PWUD face a higher risk of revocation of their probation/parole status as compared to non-PWUD [3]. One of the many downstream consequences of CLS-involvement is an increased risk of overdose [4, 5]. Multiple studies have documented that people who are on probation/parole experience a heightened risk of nonfatal and fatal overdoses [6–8].

Following release from incarceration, PWUD face a myriad of challenges in regards to their drug use, including decreased tolerance, and barriers to accessing harm reduction resources and healthcare [9, 10], which has resulted in high rates of overdose for individuals recently released from incarceration [11–13]. Combined with factors such as difficulties finding employment and housing, PWUD with a history of CLS-involvement frequently encounter challenges in accessing resources to protect themselves and others from overdose [14–16]. As a result of criminalization, fears of rearrest, reincarceration, and over-policing, some PWUD tend to use in ways that increase overdose risk in order to decrease visibility to law enforcement–such as using alone in private settings due to fear of arrest and rushing their use [2, 17, 18]. These factors are also key barriers to accessing harm reduction services [2, 17, 18].

Law enforcement involvement can heavily impact health outcomes and service access among PWUD. Many studies have demonstrated that real or perceived involvement of law enforcement is often one of the primary barriers for people seeking harm reduction, drug treatment, and recovery support services [19–23]. For example, despite implementation of Good Samaritan Laws (which provide some legal protections for people who call 911 in the event they witness an overdose), many PWUD still identify fear of contact with police and breaching probation or parole as primary reasons for not calling emergency medical services after witnessing an overdose [24]. Additionally, in Boston, utilization of a low-barrier substance use disorder service dropped during increased police activity in the immediate vicinity of the program, and subsequently rose after police activity declined, suggesting PWUD will be less likely to engage in services due to police presence [23]. In general, the literature suggests that police presence in spaces where PWUD can access harm reduction and other such services can reduce engagement.

Relative to the rest of the United States, the state of Rhode Island has lower rates of incarceration (289 per 100,000), but very high rates of people on probation and parole (664 per 100,000) [25]. In recent years, particular attention has been paid to reducing the risk of overdose among people who are leaving incarceration and those experiencing probation or parole [21,22]. In order to decrease rates of overdose among this marginalized population, it is essential to explore other novel and accessible overdose prevention methods.

Rhode Island has an extensive and unique history of pioneering programs to support PWUD and is actively investing in harm reduction programs to help prevent overdoses [26]. For example, the Rhode Island Department of Corrections (RIDOC) established the first statewide medication for opioid use disorder (MOUD) program within the prison system where those incarcerated are able to access treatment and receive linkage to care post-release [27, 28]. Rhode Island was also the first state to legalize the distribution and use of fentanyl test strips, used to help prevent overdose deaths [29]. In 2021, the Rhode Island General Assembly passed legislation that allows for the opening of overdose prevention centers (OPCs) [30], which are places where people can bring pre-obtained drugs to use under supervision of trained staff who can intervene in the event of an overdose. OPCs are an internationally recognized approach to overdose prevention and HIV prevention that has been implemented in Canada, Australia, and several European countries [31]. In New York City, OPCs have found success in preventing overdoses among PWUD in a US setting while also providing harm reduction, housing, and health services [32, 33]. Previous research suggests high willingness to use OPCs among people who inject drugs, although fewer studies have examined OPC use willingness among those who smoke, snort, or use via other methods despite a high number of fentanyl-related overdoses occurring among non-injectors in the US [34–38]. This study builds on this prior research in examining the effect of probation or parole status on people who smoke, snort, or use via other methods on OPC use willingness, which is important to investigate given that OPCs in Rhode Island are required by statute to have facilities to support supervised inhalation [39]. In addition, smoking rooms are available at OPCs in New York City and are increasingly common in other jurisdictions [40, 41]. While there appears to be high willingness to use OPCs among other racialized and surveilled populations [42], it is important to understand how probation or parole status may impact willingness to use OPCs.

As Rhode Island prepares to open its first OPC—which will be among the first in the country—it is essential to understand OPC use willingness among PWUD who are on probation or parole, as this subgroup is at higher risk of an overdose than their peers without a history of CLS-involvement [5, 12, 30]. Furthermore, given that extensive prior literature has identified fear of arrests to be a primary barrier to accessing overdose prevention centers [36, 37, 42], it is essential to explore how this may impact the willingness of people who are CLS-involved to use an OPC. Therefore, the goal of this study was to determine the association between probation and parole status with willingness to use an OPC among PWUD in Rhode Island. A secondary objective included exploring associations between willingness to use an OPC and other sociodemographic and drug use factors of interest. We hypothesized that participants on probation and parole would be less likely to report willingness to use an OPC in light of potential concerns about law enforcement involvement, probation or parole violations, and arrests.

## Methods

The Rhode Island Prescription and Illicit Drug Study (RAPIDS) is a randomized clinical trial to study the efficacy of a fentanyl overdose prevention intervention [43]. The study recruited 509 PWUD between September 2020 and February 2023. RAPIDS had the following inclusion criteria: (1) currently lived in Rhode Island, (2) were aged 18–65 years old, (3) able to complete a survey in English, and (4) use of prescription pills bought on the street, crack cocaine, powder cocaine, crystal methamphetamine, and/or heroin in the 30 days prior to recruitment, or any injection drug use in the 30 days prior to recruitment. Notably, at the time of survey development, heroin was the main opioid in the drug supply [43]. However, to reflect changes in the drug supply [44], fentanyl use was also included as part of the study inclusion criteria. Participants were recruited using the following methods: posting flyers at bus stops and various locations around local urban areas; utilizing Rhode Island Public Transport Authority bus ads; through word-of-mouth communication; and recruitment at syringe service programs (SSPs) in Rhode Island. Participants completed research visits at the Brown University School of Public Health or at SSPs in Woonsocket and Pawtucket, Rhode Island. While the RAPIDS trial involves prospective follow-up through 12 months, only baseline data were utilized for this cross-sectional analysis. Surveys were conducted by trained staff and ranged between 60 and 90 min depending on participant responses. Participants were compensated $35 for their time for the baseline survey. The RAPIDS study was approved by the Brown University Institutional Review Board.

The primary dependent variable for this analysis was willingness to use an OPC, which was assessed by the following item: “Some other countries have Supervised Consumption Rooms, which are places where people can legally bring their own drugs, get supplies like clean needles, and use their drugs in front of staff in case they overdose. If there was a legal service you could go to for free to use drugs safely indoors, would you use this service? (Yes/No).” This item was assessed and analyzed as a binary variable.

The primary independent variable explored in this study was probation or parole status, which was assessed through two separate items: “Have you ever been on probation or parole? (Yes/No);” and if “yes” to this item then, “Are you currently on probation or parole? (Yes/No).” A 3-level variable to measure CLS-involvement was created including the following mutually exclusive groups: (1) people who have never been on probation or parole; (2) people who have lifetime experience of being on probation or parole but are not currently; and (3) people who reported being currently on probation or parole at baseline assessment. Participants who chose to answer “Don’t Know/Refuse” for the primary dependent (n = 21, 4%) or the primary independent (n = 6, 1%) variables were excluded from this analysis, leaving a final analytic sample size of 482 (95% of the total sample).

Other variables assessed as covariates of interest included the following sociodemographic characteristics: biological sex at birth, self-reported gender identity (dichotomized for this analysis as cisgender vs. transgender or other), age (by year), race (white, Black, bi/multi-racial, other), ethnicity (Hispanic/Latine, non-Hispanic/Latine), and current homelessness. Drug use behaviors and patterns were also examined including regular use of crack cocaine, powder cocaine, and heroin in the past month; regular use of fentanyl in the past month; regular injection drug use in the past month; regular non-injection drug use in the past month; regular concurrent drug use in the past month (see details below); drug dealing in the past month; current use of opioid agonist therapy (OAT); history of seeing someone else overdose; and lifetime overdose history. For all drug use behaviors, regular use was operationalized as ≥ 4 days of use in the past month. For regular use of crack cocaine, powder cocaine, and heroin, the survey collected the exact number of days that the participant had used in the last 30 days (0 days ≤ n ≤ 30 days). This included both non-injection drug use and injection drug use separately. Regular drug use variables were operationalized by summing days of non-injection drug use and days of injection drug use for the following drugs to determine whether or not a participant regularly used the following substances: crack cocaine, powder cocaine, and heroin [45]. For regular use of fentanyl, we also enumerate missing data (i.e., those who indicated “don’t know” or declined to respond) to capture participants who may have been unsure about presence of fentanyl in their drugs [37–39]. Regular fentanyl use was operationalized per the following answer choices: “multiple times per day,” “every day,” “at least every week,” “once or a couple of times,” or “never” in the last 30 days. These data were collected separately from other substance use due to this study’s focus on implementation of fentanyl test strips in detection of contamination of fentanyl in other illicit substances, as well as to account for the fact that fentanyl use was not always known at the outset of drug use [43]. To best approximate regular use as defined earlier as ≥ 4 days of use in the past month, regular use of fentanyl was operationalized as “multiple times per day,” “every day,” or “at least every week.” For regular injection drug use and for regular non-injection drug use, the following substances were included: crack cocaine, powder cocaine, heroin, crystal methamphetamine, psychedelics, club drugs, non-prescribed prescription opioids, non-prescribed benzodiazepines, and non-prescribed methadone or buprenorphine. Regular non-injection drug use was defined as ≥ 4 days of use including the following methods of usage: snorting, smoking, swallowing or any other use (not including injection use). Regular concurrent drug use was operationalized as regular use of two or more drugs in the past 30 days including the same list of substances involved in regular drug use. Current OAT was defined as self-reported active enrollment in either methadone or buprenorphine treatment as prescribed from a provider.

A five-point Likert scale (i.e., strongly agree, agree, neutral, disagree, strongly disagree) was utilized to measure participant attitudes and preferences involving drug use. Self concern about overdose was operationalized to include participants who answered “strongly agree” and “agree” to the statement, “I am concerned about overdose.” Preference for using fentanyl or drugs containing fentanyl was operationalized to include participants who answered “strongly agree” and “agree” to the statement, “I prefer using fentanyl or drugs that have fentanyl in them.” Similarly, concern about using fentanyl or drugs with fentanyl was also operationalized to include participants who answered “strongly agree” and “agree” to the statement, “I am concerned about my drugs having fentanyl in them.”

Descriptive statistics were employed for all sociodemographic and substance use-related variables for the analytic sample. Bivariable associations were assessed using Pearson’s chi-square and Wilcoxon rank-sum tests for categorical and continuous variables, respectively. Fisher’s exact test was used for categorical variables where cell counts were less than 5. Finally, modified Poisson regression models were used to estimate unadjusted and adjusted prevalence ratios. The selection of covariates included in the final adjusted model was based on prior literature surrounding OPC willingness and considerations for OPC planning [35–37, 42]. For example, in Park et al. 2019, greater willingness to use an OPC was associated with gender, race, and fentanyl preference, while recent overdose was associated with less willingness to use OPC. Due to the distribution of overdose history among our sample (54% with lifetime overdose, and 14.5% with overdose in the last month), lifetime overdose history was selected to be in the model. For this model, fentanyl was chosen to be included due to its prevalence in the drug supply, and heroin use was intentionally excluded due to risk of collinearity with fentanyl use given the rapid rise of fentanyl use among heroin users in New England [46]. Further variables were included to provide insight in regards to the potential future of an OPC for PWUD in Rhode Island. Variance inflation factors (VIFs) were also calculated to assess collinearity, and variables demonstrating moderate or strong collinearity were excluded. Two-sided *p*-values were used for all variables and were considered statistically significant at *p* < 0.05.

## Results

Among 482 participants who had complete data for the primary outcome and exposure, 24% (n = 116) had never been on probation/parole, 44% (n = 212) were not currently on probation/parole but had lifetime experience, and 32% (n = 154) were currently on probation/parole. Overall, the majority of the sample was male (67%) and cisgender (97%), and the median age was 43 years old (interquartile range [IQR] 35, 53). In the sample, 56% of the sample identified as white, 19% identified as Black, 13% identified as bi/multiracial, and 12% identified as another race not previously listed. Approximately 44% of the sample were recruited through local harm reduction organizations such as Project Weber/RENEW and Safe Haven. The most frequently reported drug used regularly was crack cocaine (61%), and 27% of the analytical sample reported any regular injection drug use (see Table 1). The sample had a high proportion (88%) of people reporting substance use through non-injection use. The vast majority (86%) had seen someone overdose in their lifetime, and 54% reported having ever overdosed themselves. 25% of the sample was actively receiving OAT. Additional sociodemographic, drug use patterns, and drug use perception characteristics are presented in Table 1.

**Table 1:** Sociodemographic, drug use patterns, and drug use perception characteristics of 482 people who use drugs compared with willingness to use an OPC

Characteristic (n)	Overall n = 482 (%)	No, willingness to use OPC n = 138 (29%)	Yes, willingness to use an OPC n = 344 (71%)	*p*-value
Probation/parole status				0.24
Never been on probation/parole	116 (24%)	33 (24%)	83 (24%)	
Not currently on probation/parole	212 (44%)	68 (49%)	144 (42%)	
Currently on probation or parole	154 (32%)	37 (27%)	117 (34%)	
Sociodemographics				
Sex at birth				0.12
Male	322 (67%)	85 (62%)	237 (69%)	
Female	160 (33%)	53 (38%)	107 (31%)	
Gender identity^a^				1.00
Cisgender	464 (97%)	133 (97%)	331 (97%)	
Transgender or other	16 (3%)	4 (3%)	12 (3%)	
Age, median (IQR)	43 (35, 53)	45 (34, 56)	42 (35, 51)	0.07
Race^a^				0.33
White	269 (56%)	70 (52%)	199 (58%)	
Black	90 (19%)	32 (24%)	58 (17%)	
Bi/multi-racial	62 (13%)	16 (12%)	46 (13%)	
Other	58 (12%)	18 (13%)	40 (12%)	
Ethnicity^a^				0.82
Non-Hispanic/Latine	383 (80%)	110 (80%)	273 (79%)	
Hispanic/Latine	98 (20%)	27 (20%)	71 (21%)	
Homelessness^a^				< 0.01^b^
No	231 (48%)	80 (58%)	151 (44%)	
Yes	250 (52%)	58 (42%)	192 (56%)	
Drug use behaviors and patterns				
Regular use of crack cocaine^c^				< 0.01^b^
No	188 (39%)	71 (51%)	117 (34%)	
Yes	294 (61%)	67 (49%)	227 (66%)	
Regular use of powder cocaine^c^				0.02^b^
No	352 (73%)	111 (80%)	241 (70%)	
Yes	130 (27%)	27 (20%)	103 (30%)	
Regular use of heroin^c^				< 0.01^b^
No	329 (68%)	114 (83%)	215 (63%)	
Yes	153 (32%)	24 (17%)	129 (38%)	
Regular use of fentanyl^e^				< 0.01^b^
No	323 (67%)	110 (80%)	213 (62%)	
Yes	131 (27%)	17 (12%)	114 (33%)	
DK/R	28 (6%)	11 (8%)	17 (5%)	
Regular injection drug use^c^				< 0.01^b^
No	352 (73%)	121 (88%)	231 (67%)	
Yes	130 (27%)	17 (12%)	113 (33%)	
Regular non-injection drug use^c^				0.21
No	56 (12%)	20 (15%)	36 (10%)	
Yes	426 (88%)	118 (85%)	308 (90%)	
Regular concurrent drug use^cd^				< 0.01^b^
No	232 (48%)	93 (67%)	139 (40%)	
Yes	250 (52%)	45 (33%)	205 (60%)	
Drug dealing in the past month^a^				< 0.02^b^
No	358 (75%)	113 (82%)	245 (72%)	
Yes	120 (25%)	24 (18%)	96 (28%)	
Opioid agonist therapy^a^				0.01^b^
No	363 (75%)	114 (83%)	249 (72%)	
Yes	118 (25%)	23 (17%)	95 (28%)	
Have you ever seen someone overdose?^a^				< 0.01^b^
No	68 (14%)	30 (22%)	38 (11%)	
Yes	412 (86%)	108 (78%)	304 (89%)	
Experience lifetime overdose^a^				< 0.01^b^
No	219 (46%)	81 (60%)	138 (40%)	
Yes	258 (54%)	54 (40%)	204 (60%)	
Attitudes and preferences ance use				
Self-concern of overdose^a^				0.02^b^
No	195 (41%)	67 (49%)	128 (37%)	
Yes	286 (59%)	71 (51%)	215 (63%)	
Preference using fentanyl or drugs that have fentanyl^a^				0.02^b^
No	407 (85%)	125 (91%)	282 (82%)	
Yes	72 (15%)	12 (9%)	60 (18%)	
Concern about drugs containing fentanyl				0.17
No	133 (28%)	44 (32%)	89 (26%)	
Yes	348 (72%)	93 (68%)	255 (74%)	

^a^Responses with missing data were excluded

^b^*p* < 0.05

^c^Regular use is operationalized as ≥ 4 days of use in the last month

^d^Regular concurrent drug use is measured to be regular use of two or more of the following drugs: crack cocaine, powder cocaine, heroin, crystal methamphetamine, psychedelics, club drugs, non-prescribed prescription opioids, non-prescribed benzodiazepines, and non-prescribed methadone or buprenorphine

^e^Regular fentanyl use is operationalized as “multiple times per day,” “every day,” or “at least every week” in the last 30 days. Non-regular fentanyl use is operationalized as “Never” or “Once or a couple of times.”

In bivariate analyses comparing those who were willing to use OPCs (71%, n = 344) to those who were not (29%, n = 138), we found no significant difference in willingness to use an OPC with probation or parole status (global *p* = 0.24). Notably, we identified no significant sociodemographic differences by OPC willingness, except that those who were willing to use an OPC were significantly more likely to be homeless (56% vs. 42%;* p* < 0.01). Beyond sociodemographic characteristics, variables positively and significantly (all *p* < 0.05) associated with willingness to use an OPC included: regular use of crack cocaine, powder cocaine, heroin, and fentanyl; regular injection drug use; regular concurrent drug use; drug dealing in the past month; use of OAT; ever witnessed an overdose; experienced lifetime overdose; self-concern of overdose; and preference using fentanyl or drugs that have fentanyl.

In both unadjusted and adjusted modified Poisson regression models, probation/parole status was not significantly associated with willingness to use an OPC. As compared to those who had never been on probation or parole (reference), neither those with a lifetime history of probation/parole (unadjusted prevalence ratio [uPR] 0.95; 95% confidence interval [CI] 0.82, 1.10; adjusted prevalence ratio [aPR] 0.95; 95% CI 0.82, 1.10), nor those who were currently on probation/parole (uPR 1.06; 95% CI 0.92, 1.23; aPR 0.93; 95% CI 0.80, 1.09) significantly differed in willingness to use an OPC. In the final adjusted model, lifetime overdose history (aPR 1.16; 95% CI 1.02, 1.31) and regular use of crack cocaine (aPR 1.18; 95% CI 1.01, 1.37) were positively associated with willingness to use an OPC (see Table 2).

**Table 2:** Modified Poisson regression of the effect of probation or parole and other characteristics on willingness to use an OPC in Rhode Island of 482 PWUD from September 2020 to February 2023

Characteristic	Yes, willingness to use OPC (n = 344) (71.4%)			
	Unadjusted prevalence ratio	*P*-value	Adjusted prevalence ratio	*p*-value
Probation/parole status				
Never been on probation/parole	Ref	–	Ref	–
Not currently on probation/parole	0.95 (0.82, 1.10)	0.49	0.95 (0.82, 1.10)	0.47
Currently on probation or parole	1.06 (0.92, 1.23)	0.42	0.93 (0.81, 1.09)	0.38
Sociodemographics				
Sex at birth				
Male	Ref	–	Ref	–
Female	0.91 (0.80, 1.03)	0.14	0.93 (0.82, 1.05)	0.25
Gender identity				
Cisgender	Ref	–	–	–
Transgender	1.05 (0.79, 1.40)	0.73	–	–
Age (per year)	1.00 (0.99, 1.00)	0.12	1.00 (0.99, 1.00)	0.39
Race				
White	Ref	–	Ref	–
Black	0.87 (0.74, 1.03)	0.11	0.98 (0.83, 1.16)	0.84
Bi/multi-racial	1.00 (0.85, 1.18)	0.97	1.02 (0.88, 1.19)	0.79
Other	0.93 (0.77, 1.12)	0.46	1.01 (0.85, 1.20)	0.98
Ethnicity				
Non-Hispanic/Latine	Ref	–	–	–
Hispanic/Latine	1.02 (0.89, 1.17)	0.82	–	–
Current homelessness				
No	Ref	–	Ref	–
Yes	1.17 (1.05, 1.32)	0.01^a^	1.09 (0.98, 1.23)	0.13
Drug use behavior				
Regular use of crack cocaine^b^				
No	Ref	–	Ref	–
Yes	1.24 (1.09, 1.41)	< 0.01^a^	1.18 (1.01, 1.37)	0.03^a^
Regular use of powder cocaine^b^				
No	Ref	–	Ref	–
Yes	1.16 (1.03, 1.30)	0.01^a^	1.00 (0.88, 1.12)	0.95
Regular use of heroin^b^				
No	Ref	–	–	–
Yes	1.29 (1.16, 1.43)	< 0.01^a^	–	–
Regular injection drug use^b^				
No	Ref	–	Ref	–
Yes	1.32 (1.20, 1.47)	< 0.01^a^	1.07 (0.94, 1.21)	0.33
Regular non-injection drug use^b^				
No	Ref	–	Ref	–
Yes	1.12 (0.92, 1.38)	0.26	0.89 (0.69, 1.15)	0.39
Regular concurrent drug use^c^				
No	Ref	–	Ref	–
Yes	1.37 (1.21, 1.54)	< 0.01^a^	1.16 (0.98, 1.36)	0.08
Regular fentanyl use^d^				
No	Ref	–	Ref	–
Yes	1.32 (1.19, 1.46)	< 0.01^a^	1.08 (0.96, 1.22)	0.18
DK/R	0.92 (0.68, 1.25)	0.60	1.01 (0.75, 1.37)	0.95
Drug dealing in the past month				
No	Ref	–	–	–
Yes	1.17 (1.04, 1.31)	< 0.01^a^	–	–
Opioid agonist therapy				
No	Ref	–	Ref	–
Yes	1.17 (1.05, 1.31)	< 0.01^a^	1.07 (0.95, 1.21)	0.29
Have you ever seen someone overdose?				
No	Ref	–	–	–
Yes	1.32 (1.06, 1.64)	0.01^a^	–	–
Experience lifetime overdose				
No	Ref	–	Ref	–
Yes	1.25 (1.11, 1.41)	< 0.01^a^	1.16 (1.02, 1.31)	0.02^a^
Attitudes and preferences				
Self-concern of overdose				
No	Ref	–	–	–
Yes	1.15 (1.01, 1.29)	0.03^a^	–	–
Preference using fentanyl or drugs that have fentanyl				
No	Ref	–	–	–
Yes	1.20 (1.06, 1.36)	< 0.01^a^	–	–
Concern about drugs containing fentanyl				
No	Ref	–	–	–
Yes	1.10 (0.96, 1.25)	0.19	–	–

^a^*p* < 0.05

^b^Regular use is operationalized as ≥ 4 days of use in the last month

^c^Regular concurrent drug use was measured to be regular use of two or more drugs

^d^Regular fentanyl use is operationalized as “multiple times per day,” “every day,” or “at least every week” in the last 30 days. Non-regular fentanyl use is operationalized as “Never” or “Once or a couple of times.”

## Discussion

This study examined the association between probation or parole status with willingness to use an OPC among PWUD in Rhode Island. Contrary to our primary hypothesis, we found no association between probation and parole status with willingness to use OPCs. The results of this paper contrast findings from past research, which has demonstrated that there are potential barriers to willingness to use an OPC for some PWUD due to concerns of arrest while accessing or leaving the facility [36, 42]; this prior research suggests that OPC use willingness may be lower among CLS-involved persons for these reasons. This study builds on previous research as our population includes a high proportion of PWUD by non-injection methods. Given the increased prevalence of safe inhalation rooms, there is a need for evidence to gauge willingness to use an OPC among PWUD via non-injection methods. This study also focuses on a marginalized population, specifically people who actively experience community supervision and face increased consequences of accessing harm reduction services due to violation of probation or parole. As the results of this study suggest high willingness to use an OPC, it should be noted that previous Canadian research has shown that initial interest in a supervised injection facility was strongly correlated with later use of that facility [47]. As such, this study provides evidence that many PWUD in Rhode Island, including those who use substances via inhalation and non-injection methods, may be highly likely to use said OPC site.

While PWUD who are CLS-involved experience a high risk of re-arrests [48], and therefore may have concerns about using OPCs [29], this study suggests that willingness to use an OPC may not be affected by PWUD’s probation or parole status in Rhode Island. One possible explanation for this finding is that local harm reduction agencies have developed strong trust and rapport with PWUD through direct peer support, case management, and robust engagement of people who are unstably housed [26]; PWUD are then able to receive confidential and low-threshold services with less fear of stigma or engagement with law enforcement personnel [49]. For PWUD, harm reduction workers have been identified as trusted sources in the context of their health [50], which suggests that continued expansion of other harm reduction services, including the opening of OPCs both in Rhode Island and elsewhere, are important for PWUD with histories of CLS-involvement.

All groups (i.e., never been on probation or parole, lifetime probation/parole history but not currently on probation or parole, or currently on probation/parole) had similarly high rates of willingness to use an OPC. It is important to note that many study participants were recruited directly from harm reduction organizations who work routinely with people with CLS-involvement, and thus may not have the same reticence regarding engagement with overdose prevention services. This study adds to the current literature around interest in OPCs in demonstrating that CLS-involvement does not appear to be a barrier to OPC interest. Among PWUD in Rhode Island, these data demonstrate high willingness to use an OPC across all participants despite previous and current CLS-involvement and route of drug administration.

Other correlates of increased willingness to use an OPC in our adjusted model included crack cocaine use and experiencing lifetime non-fatal overdose. Findings from the current study may have important implications for OPC engagement and accessibility among people who do not inject drugs, people who use psychostimulants, and people of color [51, 52]. Given the high willingness to use an OPC among people who use crack cocaine, this suggests that there is a need for OPCs to offer smoking spaces for people to use safely. As overdose rates continue to climb for people who use crack cocaine, people who may not have seen themselves as at risk for an overdose, are now looking for ways to keep themselves safe [51, 53–55]. Psychostimulants, like cocaine and methamphetamine, are increasingly contributing to overdose fatalities, and in particular, are driving overdose rates among Black and other people of color [56, 57]. As such, willingness to use OPCs, especially among people who use crack cocaine, has important racial equity implications. Future OPCs in the US should be accessible for people who smoke drugs, which will be required for Rhode Island’s OPC under RI Department of Health regulations [39]. Additionally, outreach should be done so that people who smoke drugs know that they can have access to OPCs. As such, given our finding that interest in using an OPC was high among people who use crack cocaine, OPCs can fill a potential need by ensuring accessibility to people who smoke drugs.

The data in the sample also suggest that PWUD who have experienced an opioid overdose will be more likely to use an OPC. This indicates that OPCs will likely be utilized by people who have been at higher risk of overdose. Similarly, previous literature suggests that overdose history is correlated with willingness to engage in harm reduction services [58, 59], suggesting that PWUD who are more willing to use OPCs may already be engaging in harm reduction practices and are the people who may most benefit from OPC utilization. As an OPC is implemented in Rhode Island, specific efforts should be made to engage PWUD who are not already connected to existing harm reduction services, including syringe services programs; CLS-involved PWUD should also be specifically engaged, such as at post-release, at OAT transition programs, or at emergency departments following non-fatal overdose.

There are some limitations to this study. First, participants in this study were enrolled over a two-year period throughout the COVID-19 pandemic, and during a time with a rapidly changing drug supply; any temporal changes that may exist in drug use patterns and perspectives were not accounted for in this study. Second, as this study was cross-sectional, the study did not account for how each individual participant’s willingness to use an OPC may have shifted over time. Third, this study relied on self-reported data; thus data are subject to recall and information bias. Fourth, some responses may have been subject to social desirability bias given that the survey was researcher-administered rather than self-administered. Additionally, many of our participants from this study were directly recruited from SSPs, and thus may be more likely to engage with future harm reduction services, including OPCs. Lastly, this study was conducted in Rhode Island, so results from this study may not be generalizable to other states or countries. While this research suggests high willingness to use OPCs regardless of CLS-involvement, future research is needed to confirm whether OPC utilization is comparable across these groups in the US context.

## Conclusions

In summary, we found no evidence of a correlation between the probation/parole status of PWUD in Rhode Island and their willingness to use an OPC. This is particularly promising given that prior research has documented that OPC use willingness is directly correlated with future OPC utilization [47]. With establishment of a new OPC in Rhode Island, further research can and should be conducted to examine the relationship between CLS-involvement and actual use of an OPC and work to ensure individuals on probation or parole are safely able to engage with the OPC.

## Data Availability

Data may be available from the authors upon reasonable request per correspondence.

## Abbreviations

PWUD: People who use drugs
CLS: Criminal legal system
OPC: Overdose prevention center
RAPIDS: Rhode Island Prescription and Illicit Drug Study
SSP: Safe syringe program
OAT: Opioid agonist therapy
IQR: Interquartile range
uAPR: Unadjusted prevalence ratio
aAPR: Adjusted prevalence ratio
CI: Confidence interval
